# MAP4K4 and IL-6^+^ Th17 cells play important roles in non-obese type 2 diabetes

**DOI:** 10.1186/s12929-016-0307-7

**Published:** 2017-01-07

**Authors:** Huai-Chia Chuang, Tse-Hua Tan

**Affiliations:** 1 Immunology Research Center, National Health Research Institutes, 35 Keyan Road, Zhunan, 35053 Taiwan; 2 Department of Pathology & Immunology, Baylor College of Medicine, Houston, Texas 77030 USA

**Keywords:** Type 2 diabetes, Non-obese type 2 diabetes, Normal-weight type 2 diabetes, Lean type 2 diabetes, MAP4K4, Th17 cells, Visceral fat accumulation

## Abstract

Obesity is a causal factor of type 2 diabetes (T2D); however, people without obesity (including lean, normal weight, or overweight) may still develop T2D. Non-obese T2D is prevalent in Asia and also frequently occurs in Europe. Recently, multiple evidences oppose the notion that either obesity or central obesity (visceral fat accumulation) promotes non-obese T2D. Several factors such as inflammation and environmental factors contribute to non-obese T2D. According to the data derived from gene knockout mice and T2D clinical samples in Asia and Europe, the pathogenesis of non-obese T2D has been unveiled recently. MAP4K4 downregulation in T cells results in enhancement of the IL-6^+^ Th17 cell population, leading to insulin resistance and T2D in both human and mice. Moreover, MAP4K4 single nucleotide polymorphisms and epigenetic changes are associated with T2D patients. Interactions between MAP4K4 gene variants and environmental factors may contribute to MAP4K4 attenuation in T cells, leading to non-obese T2D. Future investigations of the pathogenesis of non-obese T2D shall lead to development of precision medicine for non-obese T2D.

## Background

Diabetes directly causes an estimated 1.5 million deaths in 2012, according to the World Health Organization (WHO); 90% of diabetes patients have type 2 diabetes (T2D). Finding ways to combat T2D has been one of the most important medical challenges for several decades. T2D is characterized by high blood glucose levels and insulin resistance, which are caused by impaired insulin signaling in insulin-targeted tissues including the liver, muscle, and adipose tissues. Obesity is considered to be a key cause of T2D. However, 15% of T2D patients in North America are classified as non-obese (body mass index, BMI < 30) by WHO criteria, and 50% of those in Europe and Britain are also non-obese [1]. Even when the obesity criteria for Asia is adjusted from BMI ≥ 30 to BMI ≥ 27.5, more than 60% of T2D patients in Asia are still classified as having non-obese T2D [1]. While BMI > 30 is considered to be morbid obesity, the population of non-obese T2D in Asia might be underestimated. In addition, the Obesity in Asia Collaboration also reported a lower association between increasing BMI and T2D in Asians compared with that in Caucasians [2]. Thus, other factors instead of obesity may contribute to the development of non-obese T2D. Central obesity (visceral fat accumulation) was thought to be the main cause of both non-obese and obese T2D [3]; however, several recent reports using clinical data or animal models indicate that central obesity is not associated with non-obese T2D. Recently, a door has been opened to understanding of non-obese T2D and will be described below.

## Main text

### Lack of understanding of non-obese T2D

The population of non-obese or lean T2D may be underestimated around the world. Non-obese T2D patients show a significantly higher score of “not accepting diabetes” than obese patients; many slim people do not suspect they might have T2D [4]. Notably, non-obese T2D patients have both an increased risk of heart disease and a more rapid progression to insulin treatment compared to obese T2D patients [1, 5, 6]. Furthermore, “non-obese teens are falling prey to T2D,” *Asian Age* reported in 2015. We should not ignore that numerous non-obese or slim people suffer from T2D.

To date, most attention and resources have been directed toward studying obesity-induced T2D. However, the pathogenesis of non-obese T2D cannot be readily revealed using samples from human subjects from North America due to the small sample sizes of non-obese T2D patients. It also cannot be demonstrated by the “gold standard” high-fat-diet (HFD)-fed animal model, which is not suitable for studying non-obese T2D. In some cases, studies from Western countries reported potential risk factors (such as single nucleotide polymorphisms (SNPs) of insulin receptor substrate 1 (IRS-1)) for T2D [7]; however, the association between these risk factors and T2D could not be reproduced using mostly non-obese T2D patients from Asian countries such as Turkey [8], Japan, India, and Taiwan [7]. Thus, it becomes clear that disease mechanism of non-obese T2D is different from that of obese T2D [9, 10]. Furthermore, the cause-effect relationships of the risk factors in non-obese T2D cannot be demonstrated due to the lack of relevant non-obese T2D animal models. These limitations lead to slow progress in our understanding of the pathogenesis of non-obese T2D.

### The controversy of visceral fat accumulation in non-obese T2D

Central obesity as determined by increased visceral fat accumulation was thought to be a risk of T2D in both Europeans and Asians [11]. The data derived from 290 second-generation Japanese Americans with a mean age of 61.8 indicate that intra-abdominal fat is only slightly correlated with the T2D incidence (odds ratio = 1.5), while the fasting glucose, impaired glucose tolerance (IGT) at baseline, female sex, or family history of diabetes is correlated with T2D incidence (odds ratio = 2.3, 3.8, 3.1, and 1.9, respectively) under the same multivariate model [12]. In India, both central abdominal fat and visceral fat accumulation are very slightly correlated with T2D (odds ratio = 1.001 and 1.011, respectively) [13].

Over the past decade, it is controversial whether visceral fat accumulation is the sole explanation for the high prevalence of non-obese T2D in Asia and Europe [11, 14]. In earlier studies described above [12, 13], obese or non-obese individuals from Japan or India were not subgrouped for analyses; therefore, the very slight correlation (odds ratio = 1.001, 1.011, or 1.5) between visceral adiposity and T2D may be due to that the enrolled individuals also include some obese T2D patients. Importantly, a clinical study of a consortium of 18,565 European normal-weight/overweight/obese individuals from eight European countries demonstrates that the association between insulin resistance and T2D incident is independent of central obesity (waist circumferences) and obesity (BMI) [15]. Surprisingly, insulin resistance of normal-weight T2D subjects is not positively (but inversely) correlated with central obesity (gynoid fat mass, measured by dual-energy X-ray absorptiometry (DAX)), as well as BMI [15]. Consistently, another group also reported that the BMI, waist circumferences, and fat mass of non-obese T2D patients from Europe are not significantly increased compared to control individuals [16]. Thus, visceral fat accumulation is not associated with non-obese T2D. In addition, insulin resistance of all the 18,565 enrolled individuals in the above study is correlated with increased alanine transaminase or γ-glutamyltransferase levels, suggesting that insulin resistance is associated with fatty liver (hepatic steatosis) [15]. Moreover, another group analyzed fat deposition of the lean T2D (BMI = 23) or obese T2D (BMI = 33) patients from United Kingdom by magnetic resonance imaging (MRI). They found that even lean T2D patients have an increased hepatic steatosis [17]. Collectively, measurement of hepatic steatosis, instead of BMI, waist circumferences, or body fat mass, may be a predictor for non-obese or lean T2D. This concept can be further confirmed in the future using non-obese (excluding obese) Asian T2D patients. In addition, the increase of visceral fat accumulation in some non-obese T2D patients may be a consequence of either high insulin levels during insulin resistance [18] or increased inflammatory responses [19].

### MAP4K4 gene polymorphisms and non-obese T2D

Recently, several groups identified MAP4K4 (also named HGK; not to be confused with human glucokinase and human glandular kallikrein, which are named hGK) as a risk factor for lean T2D. MAP4K4 is a member of the MAP4K family kinases [20, 21]; MAP4K1 [22] and MAP4K3 [23] have been reported as important regulators of T-cell activation [24, 25]. MAP4K4 is required for cancer cell migration and is associated with cancer metastasis [26–29]. MAP4K4 also plays an important role in motility of endothelia cells [30].

The risk factor for T2D in Europe was studied using peripheral blood DNA from the Tübingen Family (TÜF) cohort (*n* = 1,769) and the EPIC-Potsdam-derived prospective case-cohort (*n* = 2,971) [31]. They found that a SNP on the *MAP4K4* locus (rs11674694) is associated with increased oral glucose tolerance test (OGTT) glucose levels, decreased insulin sensitivity, and enhanced risks of T2D [31]. In a leaner subgroup, this *MAP4K4* SNP (rs11674694) and two other *MAP4K4* SNPs (rs13003883 and rs2236936) are also associated with increased plasma levels of IL-6 but not of TNF-α [31]. Moreover, the *MAP4K4* SNPs (rs2236936 and rs2236935) are associated with reduced insulin release only in lean subjects (BMI < 25). Consistently, the association of MAP4K4 genetic variants with T2D using 1,972 samples from Chinese Han population was also reported; this population is mainly non-obese individuals (average BMI < 27.5 in both the T2D subgroup and control subgroup) [32]. The MAP4K4 SNP rs2236935 in the MAP4K4 gene is associated with T2D. In contrast, the MAP4K4 SNP rs11674694 that associates with insulin resistance and T2D in European population is not associated with T2D in Chinese population [32]. These two results [31, 32] reflect that distinct MAP4K4 SNPs are associated with T2D in different ethnic groups. In addition, using genome-wide analysis of DNA methylation, Yamada et al. reported that the *MAP4K4* promoter is hypermethylated in Japanese patients with atherosclerosis, which is one of the fatal complications associated with T2D [33]. These reports suggest that MAP4K4 dysregulation or dysfunction is associated with non-obese T2D.

### Epigenetic regulation of MAP4K4 in non-obese T2D

**Fig. 1:**
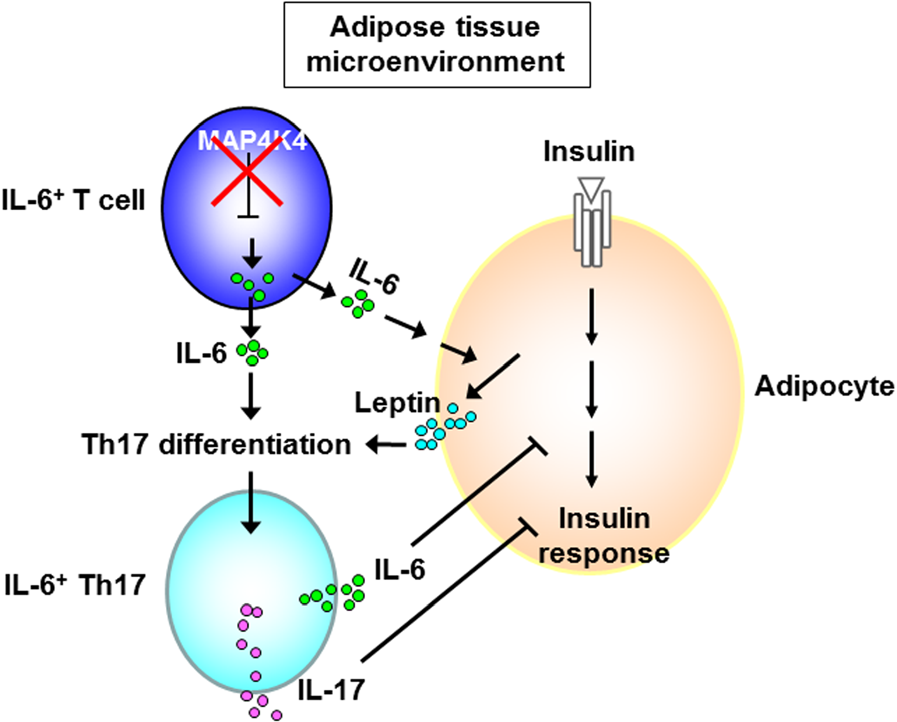
Differentiation of pathogenic IL-6^+^ Th17 cell in adipose tissue. MAP4K4 deficiency in T cells results in IL-6 overproduction. These IL-6-producing T cells infiltrate into adipose tissue. In adipose tissue microenvironment, IL-6 secreted from IL-6-producing T cells enhances leptin secretion from adipocytes. Leptin cooperates with IL-6 to promote Th17 differentiation, leading to development of pathogenic IL-6^+^ Th17 cells [34]

The pathogenesis of non-obese T2D, by which MAP4K4-deficient inflammatory T cells contribute to T2D, was recently reported [34, 35]. Normal chow diet-fed T-cell-specific MAP4K4 conditional knockout (cKO) mice spontaneously develop T2D [34]. MAP4K4 cKO T cells show TRAF2 overexpression and IL-6 overproduction. In adipose tissue of MAP4K4 cKO mice, IL-6 stimulates adipocytes to further secret the adipokine leptin (Fig. 1) [34]. IL-6-overproducing MAP4K4 cKO T cells further differentiate into pathogenic IL-6^+^ Th17 cells by a synergistic effect of leptin and IL-6 in adipose tissue (Fig. 1) [34]. These pathogenic IL-6^+^ Th17 cells then circulate to other insulin-targeted tissues (the liver and muscle), leading to insulin resistance.

The clinical significance of MAP4K4-downregulated IL-6^+^ Th17 cells is validated using purified T cells from T2D patients. Consistently, MAP4K4 levels are decreased in peripheral blood T cells from 72% drug-naive T2D patients in Taiwan [35]. In this study, all the lean T2D patients display both MAP4K4 downregulation and IL-6 overproduction in T cells [35]. The frequencies of IL-6-producing T cells are correlated with the value of insulin resistance index [35]. The frequencies of IL-6-producing T cells can be reduced by ectopically expressed MAP4K4 [35]. These findings suggest that MAP4K4 downregulation in T cells contribute to T2D. Nevertheless, due to the small sample size, the authors have not demonstrated that the percentage of obese subjects with MAP4K4 attenuation is lower than that of non-obese subjects in their study [35]. Moreover, data of demethylation treatment on the purified T cells from T2D patients demonstrate that the MAP4K4 downregulation is due to enhanced methylation on the MAP4K4 promoter [35]. Furthermore, the methylation frequencies are correlated with OGTT glucose levels independent of obesity; this correlation is particularly higher in the patient subgroup with BMI < 23 compared to those in the other two subgroups: 23 ≤ BMI < 27 (overweight) and BMI ≥ 27 (obese) [35]. Taken together, hypermethylation-mediated MAP4K4 downregulation frequently occurs in T cells of non-obese T2D patients.

### Inflammatory IL-6^+^ Th17 cells and non-obese T2D

High-fat diet (HFD) induces IFN-γ^+^ T cell population in mice [36, 37]. Besides IFN-γ^+^ T cells, inflammatory macrophages and B cells also contribute to HFD-induced T2D [37, 38]. Lack of MAP4K4 in T cells results in an induction of IL-6^+^ Th17 cell, but not macrophage or B cell, population in MAP4K4 cKO mice [34]. Unlike IFN-γ^+^ T cells, the IL17^+^ T cell population in mice cannot be induced by HFD [36]. Notably, MAP4K4-deficient IL-6^+^ Th17 cells are pathogenic cells causing spontaneous T2D in T-cell-specific MAP4K4 conditional knockout (cKO) mice, as well as in healthy recipient mice after adoptive transfer [34].

Consistently, in human drug-naïve T2D patients from Taiwan, the IL-6^+^IL17^+^ T cell population (about 20% of freshly isolated peripheral blood T cells; without any stimulation in vitro) is expanded [35]. The frequencies of the IL-6^+^IL17^+^ T cells in T2D patients from Taiwan are correlated with OGTT glucose levels but not with BMI or waist circumference [35]. Conversely, IFN-γ^+^ T cell population (about 6% of peripheral blood cells; without any stimulation in vitro) is expanded in T2D patients from the United States, while IL-17^+^ T cell population (only 0.3% of peripheral blood cells; without any stimulation in vitro) is not enhanced [39]. These findings indicate that the pathogenic IL-6^+^IL17^+^ T cell population for T2D is not induced by obesity. Collectively, these results also suggest that IL-6^+^ Th17 cells and IFN-γ^+^ T cells are important causes of non-obese T2D and obesity-induced T2D, respectively. In addition, IL-17 and IFN-γ levels from T cells of T2D patients from the Unite States can be induced in vitro by PHA or anti-CD3/CD28 stimulation [39]. The data may suggest that obese T2D patients under certain pathogenic conditions (such as infection or pollutant exposure) may induce non-obese T2D type-responses in addition to obese-type T2D. Moreover, we cannot rule out that IFN-γ^+^ T cells may cross-talk with Th17 cells in T2D patients. Thus, it is likely that the people with both obesity and MAP4K4 dysfunction may be susceptible to earlier or worse T2D.

### Prevention and therapeutic strategies for non-obese T2D

Impaired glucose tolerance (IGT) is a pre-diabetic state. The frequencies of IL-6-producting T cells from IGT patients are in between those of T2D and healthy individuals [35]. This finding suggests that examination of the IL-6^+^IL-17^+^ T cell population or MAP4K4 methylation may provide early diagnosis or even prognosis for non-obese or lean T2D.

Levels of DNA methylation can be regulated by environmental factors such as diets, pathogens, toxins, radiation, environmental hormones, air pollution, and water pH value. It is well known that air pollution (such as particulate matter, PM2.5) is associated with prevalence of T2D. Interestingly, an exposure of ozone-oxidized black carbon particles induces inflammatory T cell population in the mediastinal lymph nodes, as well as IL-6 levels in bronchoalveolar lavage fluids of wild-type mice [40]. Moreover, T-cell-specific MAP4K4 knockout mice display more severe lung inflammation than that in wild-type mice under the ozone-oxidized black carbon exposure [40].

We propose that interactions between environmental factors and *MAP4K4* polymorphisms/methylation contribute to MAP4K4 downregulation or inactivation in T cells, leading to non-obese T2D. Thus, investigating the relationship between environmental factors and MAP4K4 expression/dysfunction may help the prevention of non-obese T2D. In addition, the functional significance of the SNPs on *MAP4K4* needs to be investigated. It will also be useful to clarify whether *MAP4K4* SNPs and MAP4K4 hypermethylation co-exist or occur independently in non-obese T2D patients from different countries or different ethnic groups. Besides MAP4K4, other genes (such as signaling molecules) and environmental factors that induce pathogenic IL-6^+^ Th17 cell population may be also involved in the pathogenesis of non-obese T2D. It is also important to investigate whether other unknown pathogenic immune cell populations also contribute to non-obese T2D.

Inhibition of inflammatory T cells may be a useful approach to treatment of non-obese or lean T2D. Either IL-6 or IL-17 neutralization improves insulin sensitivity in diabetic MAP4K4 cKO mice [34]. Several studies from Japan [41] and Europe [42] indicate that rheumatoid arthritis patients receiving anti-IL-6 receptor (tocilizumab) treatment also display decreased HbA1c levels and improved insulin sensitivity. Thus, IL-6 or IL-17 blockade could be considered as a treatment for non-obese T2D. In addition, ectopic MAP4K4 expression or demethylation treatment inhibits IL-6 production in purified peripheral T cells of T2D patients [35]. Because MAP4K4 is ubiquitously expressed and MAP4K4 overexpression promotes cancer progression or metastasis [21, 26], a systemic treatment using MAP4K4 agonists may not be feasible. Thus, identification of a T-cell-specific epigenetic regulator of MAP4K4 promoter methylation may help developing a potential treatment for non-obese T2D through selective restoration of MAP4K4 in T cells.

### Interaction of T2D with rheumatoid arthritis

Rheumatoid arthritis (RA), psoriatic arthritis, or psoriasis is associated with an increased risk for T2D [43, 44]; conversely, T2D is associated with a significantly increased risk for RA [45]. The inflammatory cytokines IL-6 and IL-17 play important roles in the pathogenesis of RA, psoriatic arthritis, or psoriasis. In addition, IL-6^+^ Th17 cells are critical pathogenic cells for non-obese T2D [34, 35]. These findings suggest that the inflammatory Th17 cells may be common pathogenic cells for non-obese T2D, RA, psoriatic arthritis, and psoriasis. Thus, monitoring RA or T2D patients for inflammatory T cells will help early diagnosis of the other disease.

## Conclusions

**Fig. 2:**
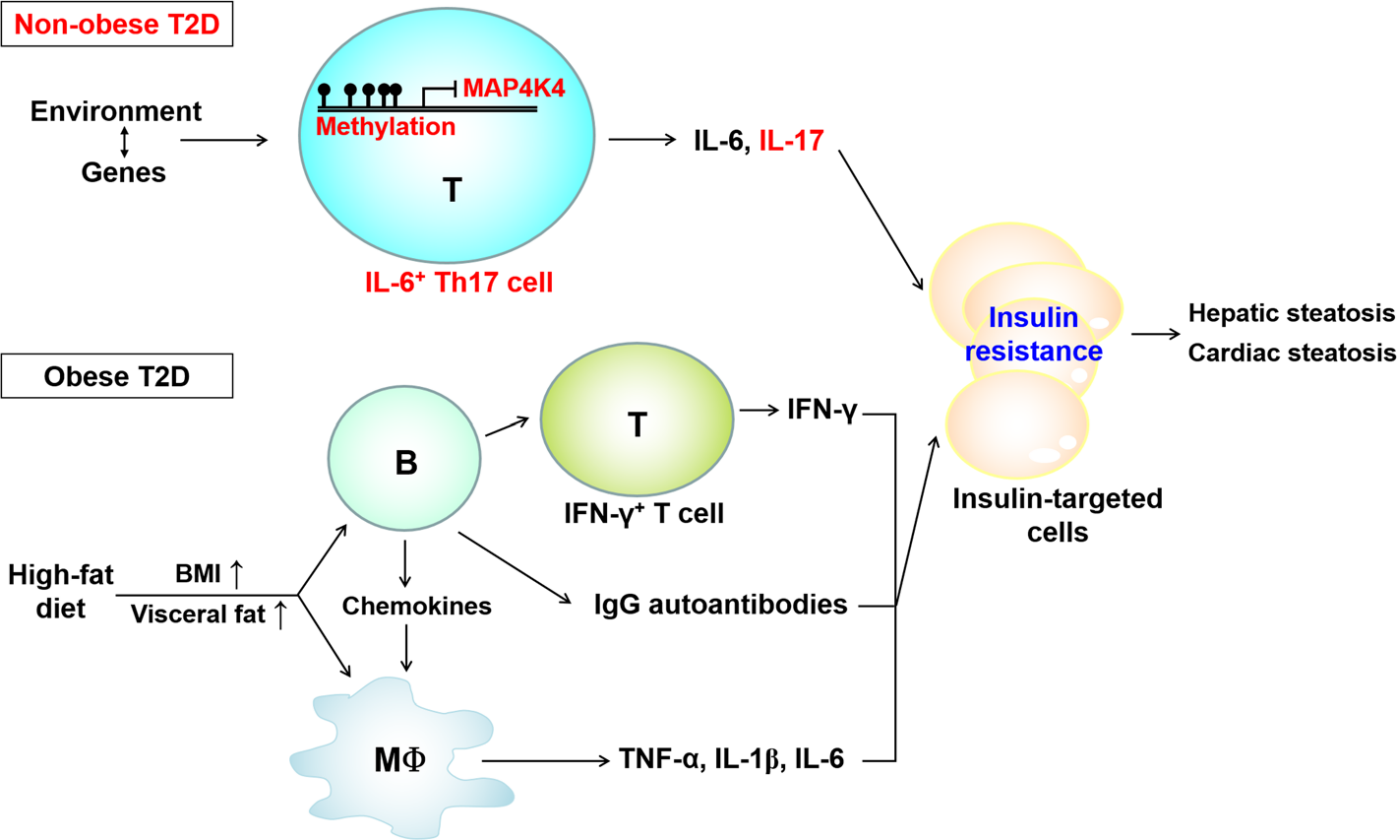
Distinct pathogenic pathways for non-obese T2D and obese T2D. For non-obese T2D, the interaction between environmental factors and genes on the MAP4K4 gene may enhance methylation of the MAP4K4 promoter, resulting in MAP4K4 downregulation. MAP4K4 downregulation in T cells subsequently results in overproduction of the proinflammatory cytokines IL-6 and IL-17, leading to insulin resistance of the insulin-targeted cells. In obese T2D, high-fat diet is a cause of obesity and visceral fat accumulation, resulting in overproduction of the proinflammatory cytokine IFN-γ from T cells [36, 37], the IgG autoantibodies from B cells [38], and the proinflammatory cytokines TNF-α, IL-1β, IL-6 from macrophages [46]. Besides macrophages, IL-6 is also produced from adipocytes, hepatocytes, muscle cells, and B cells of high-fat-diet fed mice [47]. High-fat-diet-induced B cells recruit macrophages into insulin-targeted tissues and activate T cells for IFN-γ production [38]. TNF-α and IFN-γ cause insulin resistance [46]. In both non-obese T2D and obese T2D, insulin resistance further induces hepatic and cardiac steatosis

The pathogenesis of non-obese T2D is overlooked for a long time due to the myth of the association between non-obese T2D and visceral fat accumulation. This misconception has been changed by recent publications [15, 16]. Inflammatory IL-6^+^ Th17 cells are pathogenic cells for non-obese T2D; these pathogenic T cells are caused by downregulation of MAP4K4 (Fig. 2). Moreover, the gene variants or enhanced promoter methylation of MAP4K4 may lead to the downregulation or even dysfunction of MAP4K4 in T cells. In conclusion, MAP4K4-downregulated, inflammatory Th17 cells and obesity contribute to non-obese T2D and obese T2D, respectively (Fig. 2). Furthermore, both IL-6^+^ Th17 cells and obesity may occur in the same individuals, leading to exacerbated T2D. In addition, inflammatory IL-6^+^ Th17 cells may be the underlying mechanism contributing to the association between T2D and other inflammatory diseases (such as autoimmune arthritis). Now, a door has been opened to better understanding of non-obese T2D pathogenesis. The roles of MAP4K4, IL-6^+^ Th17 cells, and visceral fat accumulation in non-obese T2D need to be further verified or clarified using clinical samples from more countries with different ethnic groups. Future investigations of the interaction between genes and environmental factors in non-obese T2D pathogenesis shall lead to development of precision medicine for non-obese T2D.

## Abbreviations

BMI: Body mass index
DAX: Dual-energy X-ray absorptiometry
HFD: High-fat diet
IGT: Impaired glucose tolerance
MAP4K4 cKO: T-cell-specific MAP4K4 conditional knockout
MAP4K4: MAP kinase kinase kinase kinase 4
OGTT: Oral glucose tolerance test
RA: Rheumatoid arthritis
SNP: Single nucleotide polymorphism
T2D: Type 2 diabetes
